# Commissioning of Varian ring & tandem HDR applicators: reproducibility and interobserver variability of dwell position offsets

**DOI:** 10.1120/jacmp.v12i4.3447

**Published:** 2011-11-15

**Authors:** Ryan McMahon, Tingliang Zhuang, Beverly A. Steffey, Haijun Song, Oana I. Craciunescu

**Affiliations:** ^1^ Department of Radiation Oncology Duke University Medical Center Durham NC

**Keywords:** brachytheraphy, high‐dose rate, tandem and ring, commissioning, offset

## Abstract

Studies have shown that source dwells within Varian's HDR CT/MR compatible ring applicators can deviate from intended positions by several millimeters. Quantifying this offset is an important part of commissioning. The aims of this study were to: 1) determine the reproducibility of the offset, 2) study the interobserver variation in the offset's measurement, and 3) quantify the dosimetric impact of the offset. Offsets were measured for four ring applicators: two 30°, one 45°, and one 60°. Dwell positions were measured five times for each ring to determine the reproducibility of source positioning. Experiments were done to compare two separate source wires, as well as different time points within a single source wire's lifecycle. Data were analyzed by three independent observers. To quantify the dosimetric impact of the offset, a treatment plan was generated using BrachyVision. The dose to point A, and the $D_{2\text{cc}}$ metric for rectum and bladder were calculated with and without the offset. For the 45° and 60° rings, measured offsets were 3.0 mm and 3.6 mm, respectively. The 30° ring showed substantial variation in distal dwell positions (maximum difference between the five experiments of 2.9 mm). Subsequent testing of a replacement ring showed an offset of 2.4 mm that was more reproducible. Offsets varied less than 1 mm between different source wires, and changed less than 1 mm over the course of a source wire's lifecycle. When comparing observers, the average range in a measurement of a dwell position was 0. 5mm (σ = 0.2 mm, max 1.3 mm). The offset resulted in dose variations to point A, bladder, and rectum of less than 1%, 2%, and 5%, respectively. Results indicate that Varian rings can show systematic and random offsets of more than 3 mm. Some can be considered defective and should be replaced. Each applicator should be individually commissioned and reproducibility should be confirmed with multiple tests.

PACS number: 87.56.‐v, 87.53.Jw

## I. INTRODUCTION

Low‐dose rate (LDR) was once the cornerstone of cervical brachytherapy, but increasing availability of remote high‐dose rate (HDR) afterloaders has lead many centers to transition to HDR.^(^ ^1^ ^–^ ^4^ ^)^ With HDR, a classical tandem and ovoid applicator can still be used, but some centers prefer to use a ring and tandem instead. Both have similar dosimetric characteristics, but the ring applicator is generally smaller than a pair of ovoids, and the larger range of dwell positions offers more flexibility for treatment planning. This flexibility for planning comes at a cost, however, as the tight curvature of the ring can be difficult for an HDR source to navigate. This curvature introduces an uncertainty in the determination of the actual dwell position of the HDR source. This positional error is the subject of this study.

Data from both end users and the manufacturer have shown that delivered dwell positions within Varian HDR ring applicators can deviate from the intended positions by several millimeters.^(^ ^5^ ^–^ ^6^ ^)^ The discrepancy arises because the source wire is much smaller than the diameter of the ring lumen through which it travels. Thus, tight curvature of an applicator can cause the source wire to take one of many possible paths. The inherent tension of the source wire can lead to fairly complex paths, and it is difficult to predict if the source will tend to follow a path that is shorter or longer than intended. In either case, if nothing is done to correct this discrepancy, there will always be an inherent difference between the planned and delivered treatments. Thus, quantifying this offset is an important part of the commissioning process for such applicators. Through customer bulletins and product notification letters,^(^ ^7^ ^–^ ^9^ ^)^ Varian has supplied a procedure for measuring the systematic component of dwell position uncertainty. Once this systematic offset is determined, a correction can be applied to treatment plans such that the delivered dwell positions will match the dwell positions used in the treatment plan.

The primary aims of this study were to: 1) determine the reproducibility (nonsystematic component) of this offset, and 2) study the interobserver variation in determining the offset. A secondary aim was to examine the dosimetric impact of applying the offset corrections. Reproducibility was determined by repetition of experiments. Interobserver variation in the analysis was quantified by comparing the results of three independent observers. The dosimetric impact was studied using a representative cervical treatment plan.

## II. MATERIALS AND METHODS

**Figure 1:**
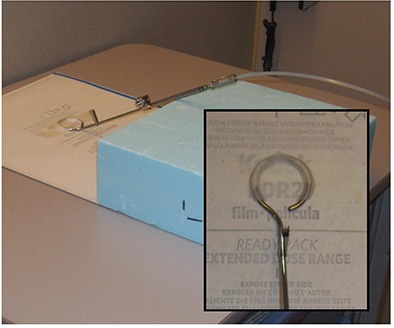
Experimental setup showing ring applicator taped to film.

**Figure 2:**
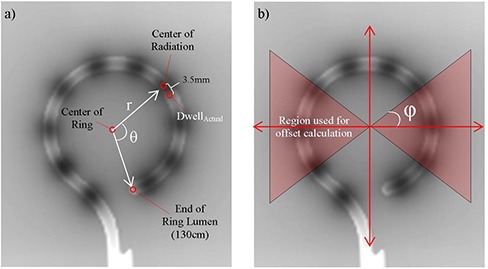
(a) Illustration of geometry of the analysis technique. Angle is measured to center of radiation, but dwell position is defined as the distal edge of the source. (b) Illustration of dwells used to determine offset. When mimicking a tandem and ovoid geometry, one could choose an angle φ less than 90° to remove anterior and posterior dwell positions from the analysis.

Four HDR ring applicators were used for this study. The rings each have a 30 mm diameter but differ in their tandem angles: two of 30°, and one each of 45° and 60°. Each ring applicator was taped, ring‐side down, to a piece of Kodak EDR2 film (Eastman Kodak Company, Rochester, NY) (Fig. 1). The film was then exposed on a conventional simulator, with ten exposures using 110 kV and 75 mAs. Since the source is taped directly to the film without buildup caps, the source‐to‐image distance will not alter the geometry of the image. With the applicator still taped to the film, an HDR plan containing ten equally spaced dwell positions (1 cm spacing) was delivered with a GammaMedPlus iX afterloader (Varian Medical Systems, Palo Alto, CA). The HDR plan used dwell times of one nominal second for a nominal 10Ci Ir‐192 source. The X‐ray exposure and dwell times were optimized to show both the applicator geometry including the lumen and the active source dwell positions on the same film (Fig. 2(a)).

### A.1 Determine offset reproducibility

The most distal dwell position used in the plan was 129.5 cm, instead of the nominal 130 cm. This is because the source can never travel past 130 cm, which is defined as the distal end of the applicator. So if the nominal 130 cm dwell position is used and the actual dwell positions are shifted distally from the planned positions, the resulting offset for the 130 cm dwell position might not be consistent with the rest of the dwell positions in the ring.

The film was scanned using a Vidar scanner (VIDAR Systems Corporation, Herndon, VA) and subsequently imported to BrachyVision (Varian Medical Systems, Palo Alto, CA) for analysis. The dwell point offset determination is illustrated in Fig. 2(a). The first step was to identify the end of the ring lumen and the origin of the circle of the ring applicator. For each dwell position, as indicated by center of the dose cloud on the film, the angle θ is obtained with the built‐in tools of BrachyVision. The arc length is calculated based on the θ value:

(1) $$ArcLength=\pi \cdot r\cdot \frac{\theta }{180}$$

where *r* is the radius of the ring. For the GammaMedPlus iX afterloader, the dwell position is defined by the distal end of the physical source, which is 3.5 mm distal to the center of the active source. Taking this 3.5 mm into account, the actual dwell position is given by:

(2) $$Dwell_{Actual}=130.0cm-(ArcLength-0.35cm)$$

$\Delta_{\text{Local}}$ is the difference between the measured and planned dwell positions, where the subscript *Local* is used to emphasize that this value is unique to each dwell position:

(3) $$\Delta_{Local}(Dwell_{Plan})=Dwell_{Plan}-Dwell_{Actual}$$

where $Dwell_{Plan}$ is the planned dwell position. Notice that if the delivered dwell position is shifted distally from the plan, then the offset $\Delta_{\text{Local}}$ will be negative. While this offset is unique to each dwell position, in practice it is easier to define a single offset $\Delta_{\text{Global}}$ that is applied to all dwell positions. As our intended application was to mimic a tandem and ovoid geometry, $\Delta_{\text{Global}}$ was determined by minimizing the squared deviation over the dwell positions that would fall within a typical ovoid geometry. In general, this can be described by defining Θ as the set of all dwell positions which fall within an angle of ±φ from the lateral dwell positions (see Fig. 2(b)). In this study, an angle $\varphi =45^{\circ }$ was used in the offset calculation to mimic a pair of ovoids. Then the global offset $\Delta_{\text{Global}}$ is calculated using a least‐square minimization over this set of dwells:

(4) $$\Delta_{Global}=x:\min\sum_{\Phi }{\left(\Delta_{Local}-x\right)}^{2}$$

The experiment outlined above was performed for four ring applicators, two source wires, and for two time points within the source wire's lifecycle. The experiments and results were organized to address the following questions:

1) Are the offsets of consecutive source‐runs consistent with each other?
2) Does the offset change over the course of the source wire's lifecycle?
3) Does the offset change with different source wires?

To address question 1, the experiment was repeated five times for each ring to determine the reproducibility of source positioning. All rings were analyzed during the same source lifecycle. These experiments were performed with a single source wire, late in the source wire's lifecycle. A linear source guide (used for daily positional QA) was used as a control setup, since it should have little inherent positional uncertainty.

To address question 2, a 30° ring was analyzed at two time points in a source wire's lifecycle. First, a set of five experiments was performed at the time of source exchange (< 30 active wire cycles). Another set of five experiments was performed near the end of the same source wire's lifecycle (> 300 active wire cycles).

To address question 3, 45°, and 60° rings were analyzed for two source wires. For both source wires, the experiments were run late in the source wire's lifecycle. Experiments were also run with the control setup to show differences in the positional calibrations of the two source wires.

### A.2 Interobserver variation in the offset measurement

Due to the subjective nature of the analysis tasks, the measured offset could depend on the observer. To quantify the interobserver effect, the same data were analyzed by three independent observers. For each data point (1 dwell position, 1 ring, 1 experiment), the maximum difference between any two observers was recorded. We report the average and maximum of this value over all data points. This corresponded to 4 applicators × 10 dwell positions × 5 experiments = 200 data points.

### A.3 Quantification of dosimetric impact

**Figure 3:**
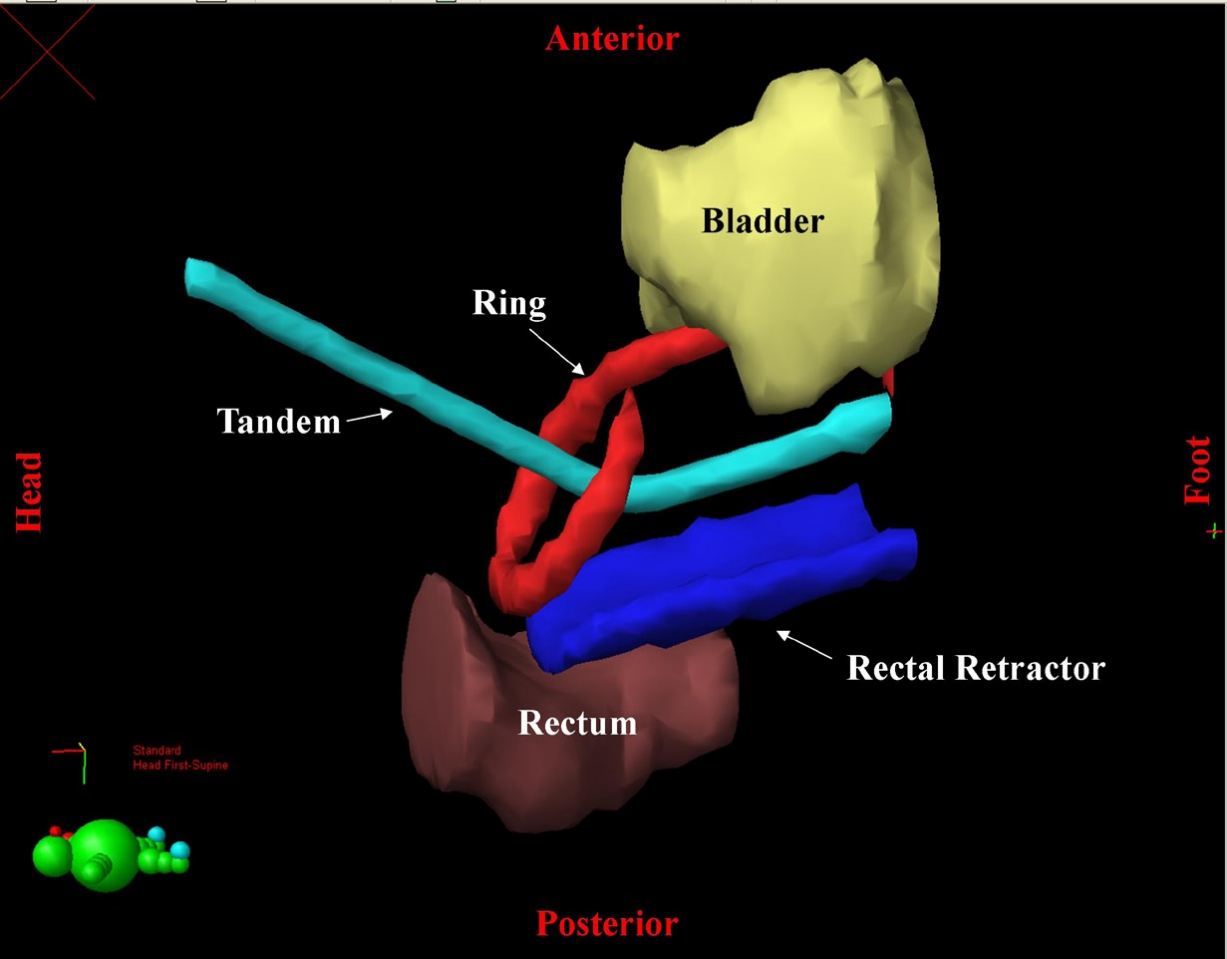
Treatment plan geometry. Bladder and rectum are contoured as critical structures. The applicator consists of a tandem, ring, and rectal retractor. Reference lines used for optimization are also shown.

To assess the dosimetric impact of the dwell point offset, a treatment plan was generated in Brachy Vision for a representative cervical implant using the 45° ring and tandem applicator. Bladder and rectum were contoured as critical structures. The tandem was loaded to the mid‐ring level, and the rings were only loaded laterally to mimic a tandem and ovoid geometry. Optimization points and lines followed ABS guidelines.^(^ ^10^ ^)^ Reference lines for optimization were placed bilaterally along the tandem and ring. Dwell positions were optimized to deliver 500 cGy to these reference lines. The treatment geometry is shown in Fig. 3.

The measured dwell positions from the 45° ring were applied to this treatment plan to estimate the dosimetric impact of the positional uncertainty. One plan was calculated without applying any offset correction. A corresponding plan was calculated using the same measured dwell positions, but the offset $\Delta_{\text{Global}}$ was used as a correction. The parameters used to compare these plans were 1) dose to point A, and 2) the maximum dose to 2 cc of the bladder or rectum $\left(D_{2\text{cc}}\right)$.^(^ ^11^ ^)^ Since the offset $\Delta_{\text{Global}}$ varied little between rings, only the 45° ring was analyzed for dosimetric purposes.

## III. RESULTS

### A.1 Reproducibility of dwell offset

**Figure 4:**
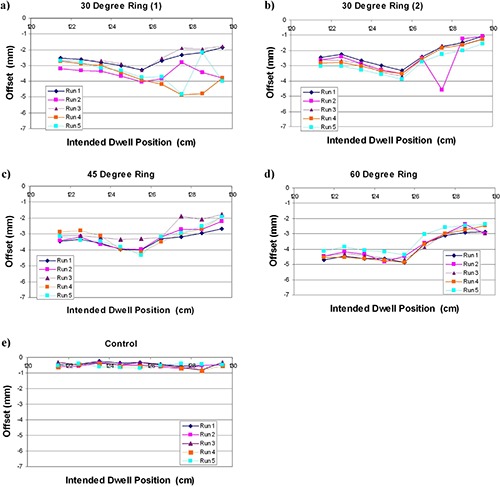
Reproducibility of dwell positions for: (a) original 30° ring, (b) replacement 30° ring, (c) 45° ring, (d) 60° ring. Negative offset corresponds to a delivered dwell position shifted distally from the planned position.

The measured offsets $\left(\Delta_{\text{Local}}\right)$ for a single observer are shown in Fig. 4. All offsets were negative, meaning the actual dwell positions were shifted distally from the planned dwell position. Table 1 reports the offset correction $\Delta_{\text{lobal}}$, range of actual measured offsets $\left(\Delta_{\text{Local}}\right)$, and maximum variation between any two experiments.

**Table 1:** Summary of offsets. The derived offset is determined via Eq. (4), with a least squared minimization for lateral dwell positions. The maximum variation is a comparison between common dwell positions amongst all five experiments.

*Applicator*	$\Delta_{Global}$ *(mm)*	*Range* $\left(\Delta_{Local}\right)$ *(mm)*	*Max Variation Between Experiments (mm)*	*Dwell Position of Max Variation (cm)*
30° (1)	−3.0	−4.8,−1.8	2.9	127.5
30° (2)	−2.4	−4.6,−1.0	2.9	127.5
45°	−3.0	−4.3,−1.8	1.3	127.5
60°	−3.6	−4.9,−2.4	0.8	126.5
Control	−0.5	−0.8,−0.2	0.4	128.5

For the 45 and 60° rings, the offset correction $\left(\Delta_{\text{Global}}\right)$ was −3.0 mm and −3.6 mm, respectively. The 30° ring had an offset correction of −3.0 mm, but showed substantial variation in the distal dwell positions between the five experiments (maximum difference in $\Delta_{\text{Local}}$ between experiments of 2.9 mm). Due to this large variability, the ring was replaced by the vendor.

**Figure 5:**
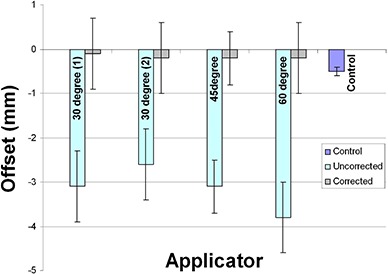
Average dwell position discrepancy with and without an offset correction (averaged over entire ring). Blue bars represent average offset without correction, and gray bars are the corresponding corrected values. Negative offset indicates a dwell position that is shifted distally from planned position. Error bars represent 1 standard deviation.

Subsequent testing of the new ring showed an offset correction $\Delta_{\text{Global}}$) of −2.4 mm that was more reproducible over the entire range of dwell positions, though a single large variation in $\Delta_{\text{Local}}$ (2.9 mm) was still seen between experiments. The 45° and 60° rings were more stable, and showed maximum variation in $\Delta_{\text{Local}}$ between experiments of 1.3 mm and 0.9 mm, respectively. Figure 5 shows the average positional offsets (averaged over the entire ring and all experiments) with and without the correction applied.

**Figure 6:**
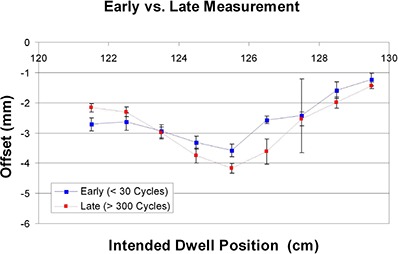
Comparison of offsets measured early and late in a source wire's lifecycle. Early measurements were done immediately after source exchange (< 30 cycles), and late measurements were done near the end of the wire's lifecycle (> 300 cycles).

The measured offset for the replacement 30° ring varied little between experiments run early and late in the source wire's lifecycle (see Fig. 6). Though the curves are not identical, the maximum discrepancy between the average offsets was 1 mm. Six of nine positions were within 0.5 mm of each other, which is comparable to the interobserver uncertainty results described in the next section. The derived offset corrections for the early and late measurements were 2.4 mm and 2.5 mm, respectively.

**Figure 7:**
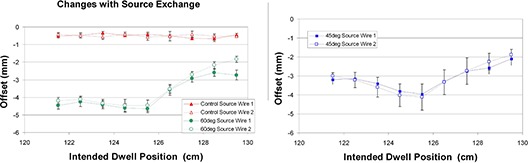
Comparison of offsets measured for two separate source wires. Measurements were done for both 45° and 60° rings. All measurements were done late in the source wire's lifecycle.

The measured offsets for the 45° and 60° rings changed little when the source wire was exchanged (see Fig. 7). Average discrepancies were again comparable to the interobserver uncertainty. For the 45° ring, the offsets at each dwell position, when averaged over all five experiments, were all within 0.4 mm of the measurements for the previous source wire.

For the 60° ring, eight of the nine average offsets were within 0.4 mm of the previous source wire, while the most distal dwell position showed a difference of 0.9 mm. For the 45° ring, the derived offset corrections $\left(\Delta_{\text{Global}}\right)$ for source 1 and 2 were both 3.0 mm. For the 60° ring, the derived offset corrections for source 1 and 2 were 3.6 mm and 3.3 mm, respectively.

### A.2 Interobserver variability

When comparing the three observers, the average range in measured dwell positions for a single data point was 0.5mm(σ=0.2mm,max=1.3 mm). Table 2 summarizes the offset corrections $\left(\Delta_{\text{Global}}\right)$ measured by each observer. For a given ring applicator, the offset corrections for all observers were within 0.2 mm of each other.

**Table 2:** Offset corrections measured by each independent observer.

*Observer*	$\Delta_{Global}$ *30° (1)*	$\Delta_{Global}$ *30° (2)*	$\Delta_{Global}$ *45°*	$\Delta_{Global}$ *60°*
1	3.0	2.4	3.0	3.6
2	3.0	2.3	3.0	3.6
3	3.1	2.3	2.9	3.4

### A.3 Quantification of dosimetric impact

**Figure 8:**
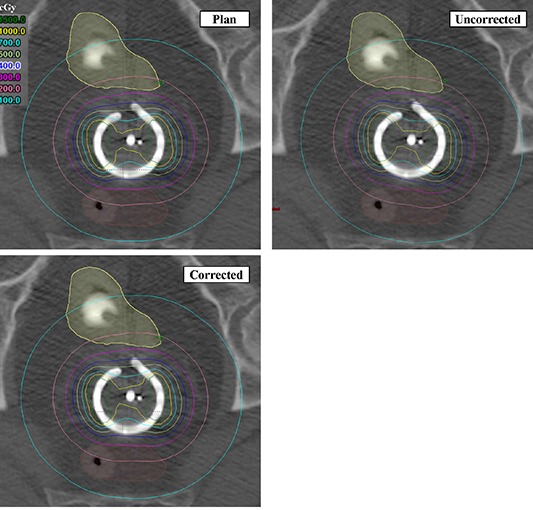
Comparison of isodose curves in the ring‐plane. These representative results are from experiment 1 with the 45° ring. Upper left shows the intended plan. Upper right shows the delivered isodose when the offset correction is not applied. Lower left shows the delivered isodose when the correction is applied.

**Figure 9:**
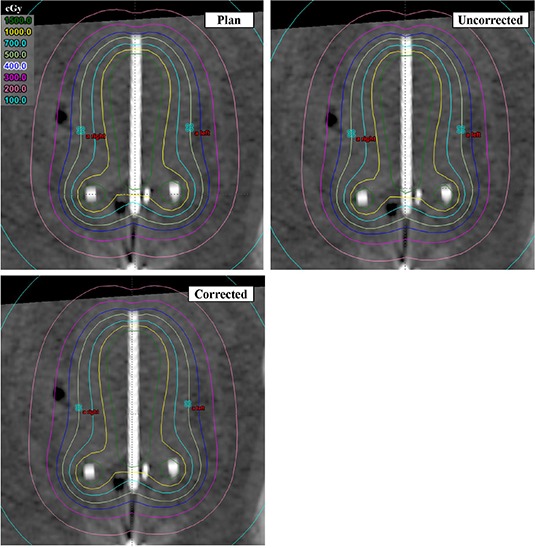
Comparison of isodose curves in the tandem‐plane. These representative results are from experiment 1 with the 45° ring. Upper left shows the intended plan. Upper right shows the delivered isodose when the offset correction is not applied. Lower left shows the delivered isodose when the correction is applied.

Figure 8 shows a comparison of isodose distributions for corrected and uncorrected treatment plans. These results are generated from the 45° ring, experiment 1, and use the measured offset data from observer 1. This figure shows that the offset produces a noticeable rotation of the isodose curves in this plane. Figure 9 compares a slice taken in the plane of the tandem. As one would expect, dosimetric differences in this plane are smaller. In Figs. 8 and 9, the image labeled ‘plan’ is the ideal treatment plan, so the ‘corrected’ plan should be a better representation of it than the ‘uncorrected’ plan. Table 3 summarizes the dosimetric comparison of corrected and uncorrected plans for all five experiments done with the 45° ring. With uncorrected plans, doses to point A varied by less than 1%. For the same uncorrected plans, the dose received by 2 cc of the bladder and rectum was within 2% and 5% of the original plan. Applying an offset correction reduced these discrepancies in all cases.

**Table 3:** Summary of dosimetric comparison between treatment plans with and without offset correction.

	*Uncorrected*			*Corrected*		
*Experiment*	*Point A Dose (Lt, Rt) (cGy)*	*Bladder* $D_{2cc}$	*Rectum* $D_{2cc}$	*Point A Dose (Lt, Rt) (cGy)*	*Bladder* $D_{2cc}$	*Rectum* $D_{2cc}$
*Plan*	*(509, 504)*	*197*	*240*	–	–	–
1	(505, 504)	200	252	(507, 504)	196	244
2	(505, 504)	198	252	(507, 504)	195	244
3	(506, 504)	198	249	(508), 503)	195	243
4	(505, 504)	198	252	(507, 504)	195	244
5	(505, 504)	198	252	(507, 504)	195	244

## IV. DISCUSSION

Determination of the dwell position offset is an important part of the commissioning process for Varian ring and tandem HDR applicators used with GammaMedPlus iX afterloaders. The same effect has also been demonstrated for VariSource afterloaders ((Varian Medical Systems, Palo Alto, CA).^(^ ^5^ ^)^ For the four rings tested here, offsets were all between 2–4 mm (delivered dwell position shifted distally from plan), which translates to an angular deviation of approximately 8°–2° within the ring. This is consistent with published data from other institutions using both GammaMed and Nucletron (Veenendaal, The Netherlands) afterloaders^(^ ^5^ ^–^ ^6^ ^)^ Most relevant to the results presented here are the offset measurements for a GammaMed afterloader by Stern and Liu^(^ ^6^ ^)^ that showed delivered dwell positions were shifted distally from planned positions by 2–6 mm for 30°, 45°, and 60° rings.

This systematic component of the offset should be measured during commissioning of the applicator so that treatment plans can be corrected if necessary. In practice, the correction $\Delta_{\text{Global}}$ can be applied in either the delivery or planning phase. To account for it in the delivery phase, the treatment plan is done without considering the offset (i.e., with nominal dwell positions), but then the delivered plan must use dwell positions equal to $\left(\text{nominal}+\Delta_{\text{Global}}\right)$. The second is to deliver a plan using nominal dwell positions, after the treatment plan is designed using dwell positions equal to $\left(\text{nominal}-\Delta_{\text{Global}}\right)$. In principle, both of these approaches are equivalent. In practice, safety considerations and the sign of $\Delta_{\text{Global}}$ will determine which procedure is more robust.

The results also indicate that distal dwell positions can potentially show up to 3 mm in variation between successive treatments. The position of maximum variation between experiments for all rings occurred within 1 cm of each other. This could indicate that the source wire can take multiple paths once it reaches this region of the ring. To determine this variability, offsets must be measured multiple times for each applicator. Two of the rings tested showed large variability between experiments. Based on this data, it is not clear whether this variability is inherent to all of the Varian ring applicators. The 45° and 60° rings did not show the same level of variability, but more experiments would have to be run to see whether this is a consequence of the limited number of samples.

Our results showed that the offset can be a complicated function of dwell position within the ring. This could indicate that the offset is not only dependent on the ring applicator, but also on the mechanics of a given source wire. One could also suspect that the mechanics of a source wire changes over its lifetime. Issues such as these are important when considering a QA program for these applicators. To address the question of how frequently these offsets should be measured, we studied the dependence on source wires, as well as the time dependence within a single source's lifecycle. The results show no substantial variation in offsets that are averaged over five experiments. The derived global offset corrections varied even less. Stern and Liu^(^ ^6^ ^)^ also reported no significant differences in offset measurements for different source wires. Based on these results, offsets can be measured at any point in a source wire's lifecycle. Moreover, this study has not recognized any variability in offset that would justify a remeasurement at each source exchange. However, we are only reporting data from two source wires, and more data is needed for a final recommendation.

The analysis requires an observer to identify: 1) the end of the ring lumen, 2) the center of the ring, and 3) the center of each delivered dwell position. In this study, the potentially subjective nature of these steps introduced little interobserver variability. When comparing three independent observers, the average range in measured dwell positions was 0.5 mm (max 1.3 mm). These differences translated into less than 0.2 mm difference in offset corrections. Based on these results, it would not be necessary to have multiple observers analyze measured data for the purposes of obtaining a statistical average.

Through technical bulletins and product notification letters, Varian has released data that suggest the dosimetric impact of the offset could be up to 20% variation in dose to critical structures. The plan evaluated in this study showed that the offsets have only a small effect on the dose to point A, bladder $D_{2\text{cc}}$, and rectum $D_{2\text{cc}}$ (less than 1%, 2%, and 5% difference, respectively). These results are expected to be representative of the scenario where a ring applicator is used to mimic a tandem and ovoid geometry, and agree well with a similar study that showed <3% variation in dose to points A/B, bladder, and rectum.^(^ ^6^ ^)^ The dosimetric discrepancies could be larger for nonconventional plans that are more conformal,^(^ ^12^ ^–^ ^13^ ^)^ particularly if anterior or posterior dwell positions are used.

Even though the dosimetric impact of the offset itself may be small, it is only one component of positional uncertainty when using ring applicators. In addition to the systematic and random fluctuations that are inherent to the ring, there can be considerable interfractional^(^ ^14^ ^–^ ^15^ ^)^ or intrafractional^(^ ^16^ ^)^ variation in the positioning of the applicator. These effects could combine to produce larger dosimetric discrepancies than shown here, particularly if a new plan is not generated for each fraction. For example, the offsets measured in this study amount to angular deviations within the ring of 8°–12°. This is potentially larger than reported interfractional angular variation in ring applicator placement.^(^ ^14^ ^)^

## V. CONCLUSIONS

Results indicate that Varian rings can show systematic and random offsets of > 3 mm. Some can be considered defective and should be replaced. Each applicator should be individually commissioned and reproducibility should be confirmed with multiple tests. Offsets varied less than 1 mm between different source wires, and changed less than 1 mm over the course of a source wire's lifecycle. Interobserver variability in the determination of the offset correction was less than 0.2 mm. Offsets translated to <5% variation in dose to critical structures, which is less than vendor‐reported results.

## ACKNOWLEDGMENTS

We would like to thank John Morrison from Varian Medical Systems for his technical advice and expertise.

## Supporting information

Supplementary Material
