# Supplementary figures and images for: Commissioning of Varian ring & tandem HDR applicators: reproducibility and interobserver variability of dwell position offsets
Source: J Appl Clin Med Phys. 2011 Nov 15;12(4):50–62. doi: 10.1120/jacmp.v12i4.3447 (PMC5718734; doi:10.1120/jacmp.v12i4.3447)

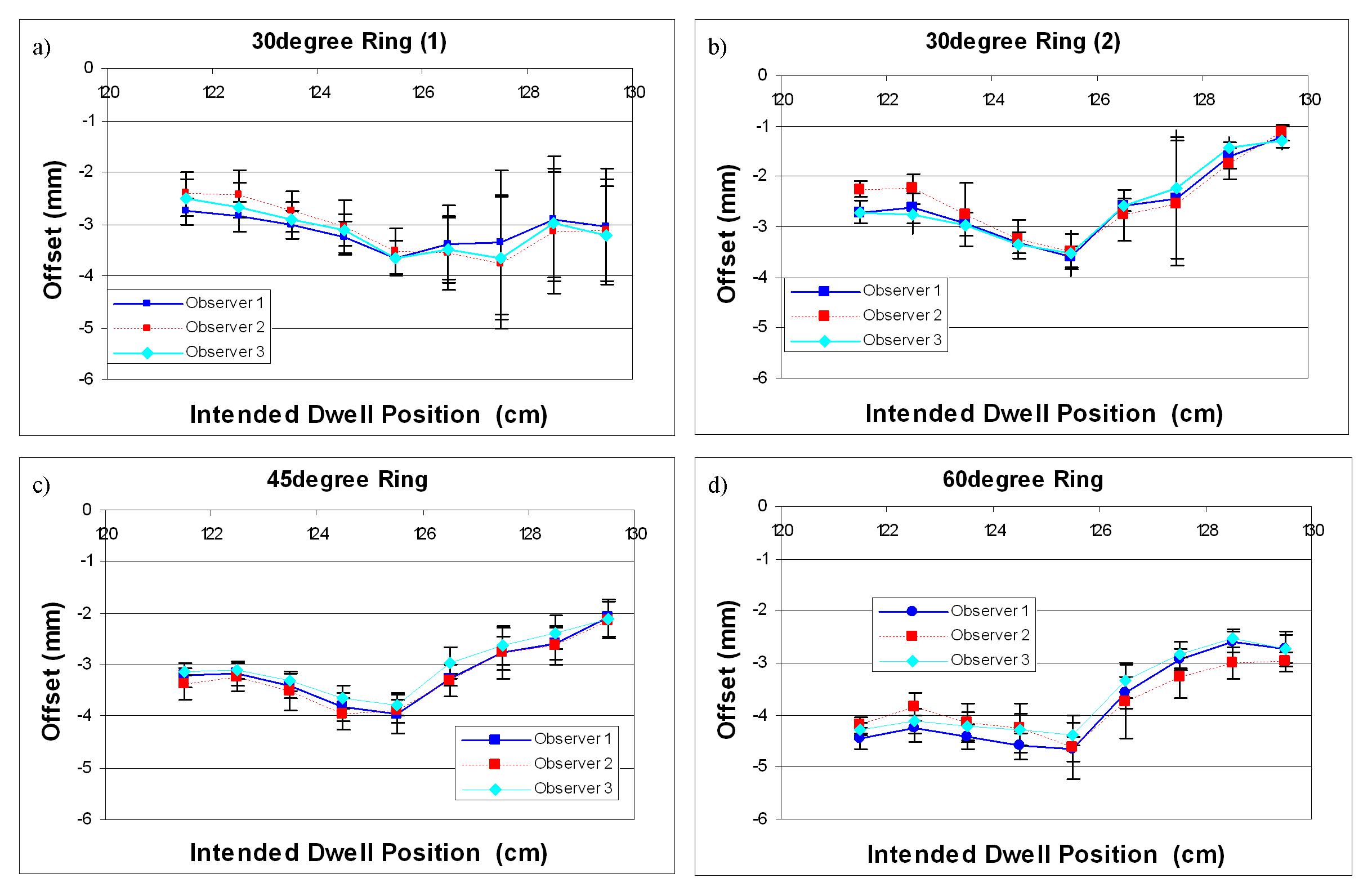

Supplement: Supplementary file 1 — Supplementary Material [file ACM2-12-050-s001.jpg]
